# Perioperative, functional, and oncological outcomes of robotic vs. laparoscopic partial nephrectomy for complex renal tumors (RENAL score ≥7): an evidence-based analysis

**DOI:** 10.3389/fonc.2023.1195910

**Published:** 2023-06-02

**Authors:** Li Wang, Jing-ya Deng, Cai Liang, Ping-yu Zhu

**Affiliations:** ^1^ Department of Urology, Affiliated Hospital of North Sichuan Medical College, Nanchong, China; ^2^ Department of Neurology, Affiliated Hospital of North Sichuan Medical College, Nanchong, China; ^3^ Department of General Medicine, The Second Affiliated Hospital of North Sichuan Medical College (Mianyang 404 Hospital), Sichuan, China

**Keywords:** partial nephrectomy, minimal invasive, laparoscopy, robotics, neoplasms

## Abstract

**Objective:**

To evaluate the current literature comparing outcomes of robotic partial nephrectomy (RPN) versus laparoscopic partial nephrectomy (LPN) treating complex renal tumors (RENAL nephrometry score ≥7).

**Methods:**

We systematically searched the Cochrane Library, PubMed, Google Scholar, EMBASE, and Scopus databases up to March 2023. Review Manager 5.4 performed a pooled analysis of the data for random effects. Besides, sensitivity and subgroup analyses to explore heterogeneity, Newcastle-Ottawa scale, and GRADE to evaluate study quality and level of evidence.

**Results:**

Eight observational studies comprising 1346 patients (RPN: 695; LPN: 651) were included in this study. Compared to LPN, RPN had a shorter operative time (OT) (weight mean difference [WMD]: -14.73 min; p = 0.0003), shorter warm ischemia time (WIT) (WMD: -3.47 min; p = 0.002), lower transfusion rate (odds ratio [OR]: 0.66; p = 0.04), shorter length of stay (LOS) (WMD: -0.65 days; p < 0.00001), lower postoperative estimated glomerular filtration rate (eGFR) change (WMD = -2.33 mL/min/1.73 m2; p = 0.002) and lower intraoperative complications (OR: 0.52; p = 0.04). No significant differences were observed between the two groups in terms of estimated blood loss (EBL) (p = 0.84), conversion to radical nephrectomy (p = 0.12), postoperative complications (p = 0.11), major complications (defined Clavien–Dindo grade 3 (p = 0.43), overall complications (p = 0.15), postoperative eGFR (p = 0.28), local recurrence (p = 0.35), positive surgical margin (PSM) (p = 0.63), overall survival (OS) (p = 0.47), cancer-specific survival (CSS) (p = 0.22) and 3-year recurrence-free survival (RFS) (p = 0.53).

**Conclusion:**

Patients with complex renal tumors (RENAL score ≥7), RPN is superior to LPN in decreasing the OT, WIT, LOS, transfusion rate, change in eGFR and the incidence of intraoperative complications while maintaining oncological control and avoiding a decline in renal function. However, our findings need further validation in a large-sample prospective randomized study.

## 1 Introduction

Currently, partial nephrectomy (PN) is the preferred treatment for clinical T1 renal masses, as its oncological results are comparable to those of radical nephrectomy (RN) (1). Laparoscopic PN (LPN) and robotic PN (RPN) are minimally invasive techniques that are frequently used to preserve the kidney. Minimally invasive surgery for PN was initially developed for small renal masses; however, its applicability has expanded to include complex renal tumors (2).

Complex Renal tumors that are difficult to treat are usually deeply rooted within the renal parenchyma, located near the center of the kidney in a vertical plane, and are situated near the renal collecting system (3). We selected the RENAL nephrometry score to quantify the anatomy of renal tumors measurably and classify the complexity of renal masses (4).

However, certain technical difficulties, such as intracorporeal suturing skills and an elevated warm ischemia time, are associated with LPN (5). Conversely, RPN provides superior manual dexterity, enhanced visualization, tremor elimination, and an ergonomic environment to augment surgeon comfort, thereby widening the applicability of minimally invasive surgery to encompass more intricate and arduous renal tumors (6). It should be noted that once a surgeon has acquired considerable experience in laparoscopic surgery, the benefits conferred by robotic assistance may not necessarily be maintained (7). Consequently, we conducted a meta-analysis to assess the safety and efficacy of RPN and LPN for the management of complex renal tumors.

## 2 Methods

This systematic review was performed according to the statement Preferred Reporting Items for Systematic Reviews and Meta-Analyses (PRISMA) (registration number: CRD42023411277) (8). The reporting items are outlined in the PRISMA checklist (**Table S1**).

### 2.1 Search strategy, selection, and data extraction

We performed a comprehensive search of medical databases, including PubMed, Scopus, EMBASE, and the Cochrane Library, limited to articles in English, using a combination of disease and intervention keywords. The search was performed up to 1 March 2023, and the search terms were as follows: [(Robotic partial nephrectomy OR Robot-assisted partial nephrectomy) AND (laparoscopic partial nephrectomy OR laparoscopic nephron-sparing surgery) AND (Kidney cancer OR Renal tumor OR Renal mass) AND (Complex OR Complexity)]. Relevant references, abstracts, and conference proceedings were also meticulously searched and inspected to avoid potential omissions.

The search strategy was constructed according to the PICOS principle to determine the studies to be included. The PICOS principle was as follows: P (patients) with a RENAL score of ≥7 who were diagnosed with complex renal tumors; I (intervention) of RPN; C (comparator) of LPN for comparison; O (outcome) of surgical parameters, renal functional and oncological outcomes; and S (study type) of both prospective and retrospective case-control studies and randomized controlled trials (RCTs). The exclusion criteria will be included when the following situations are observed: (1) lack of data for meta-analysis; (2) non-comparative studies; (3) conference abstracts, case reports, letters, and any other unpublished articles.

Two reviewers (WL and LC) distinguished the conclusive literature by eliminating duplicates, perusing abstracts at the title level, and performing a full-text audit of all incorporated studies using Endnote X9 (London, UK). A senior researcher (JY) was consulted in the case of disparities. The data extraction process was subsequently implemented, trailed by the ordering of the study data using preset Excel tables.

### 2.2 Quality assessment and risk of bias

The level of evidence was determined according to the Oxford Level of Evidence Working Group 2011. For non-RCTs, the Newcastle–Ottawa scale was used to evaluate the quality of the included studies (9), with a score of ≤5 indicating low quality, 6–7 indicating moderate quality, and 8–9 indicating high quality. The ROBINS-I tool was used to assess the risk of bias in each study (10); discrepancies, if any, were settled through negotiation.

### 2.3 Statistical analysis

Review Manager 5.0 (Oxford, UK) and Stata 14.0 (TX, USA) were used to perform this meta-analysis. A random-effects model was used to calculate the pooled weighted mean difference (WMD), odds ratio (OR), and hazard ratio (HR) with 95% confidence intervals (CIs).

The I^2^ statistic was used to determine the significance of heterogeneity, with the threshold value being I^2^ >60%. Individual participant data will be reconstructed from published Kaplan-Meier survival curves (11). Based on the conversion tables provided by Luo et al. (12) to transform raw data from the median and interquartile range or maximum and minimum values into normal distribution data, as well as McGrath et al. (13) formula to transform non-normally distributed data. Statistical significance was established at p <0.05.

Additionally, funnel plots were used to assess the publication bias of included studies (only for comparisons containing the most studies and high heterogeneity). The Begg and Egger test could not be used to perform the publication bias test because of the insufficient test power (studies <10) (14, 15). We performed sensitivity analyses to assess the reliability of our estimates using the leave-one-out method, in which the studies were sequentially eliminated from the pooled effect. However, this criterion was not applied when comparing less than three studies. Furthermore, a subgroup analysis was performed according to the design, country and sample size of the study.

## 3 Evidence synthesis

### 3.1 Baseline characteristics

**Figure 1:**
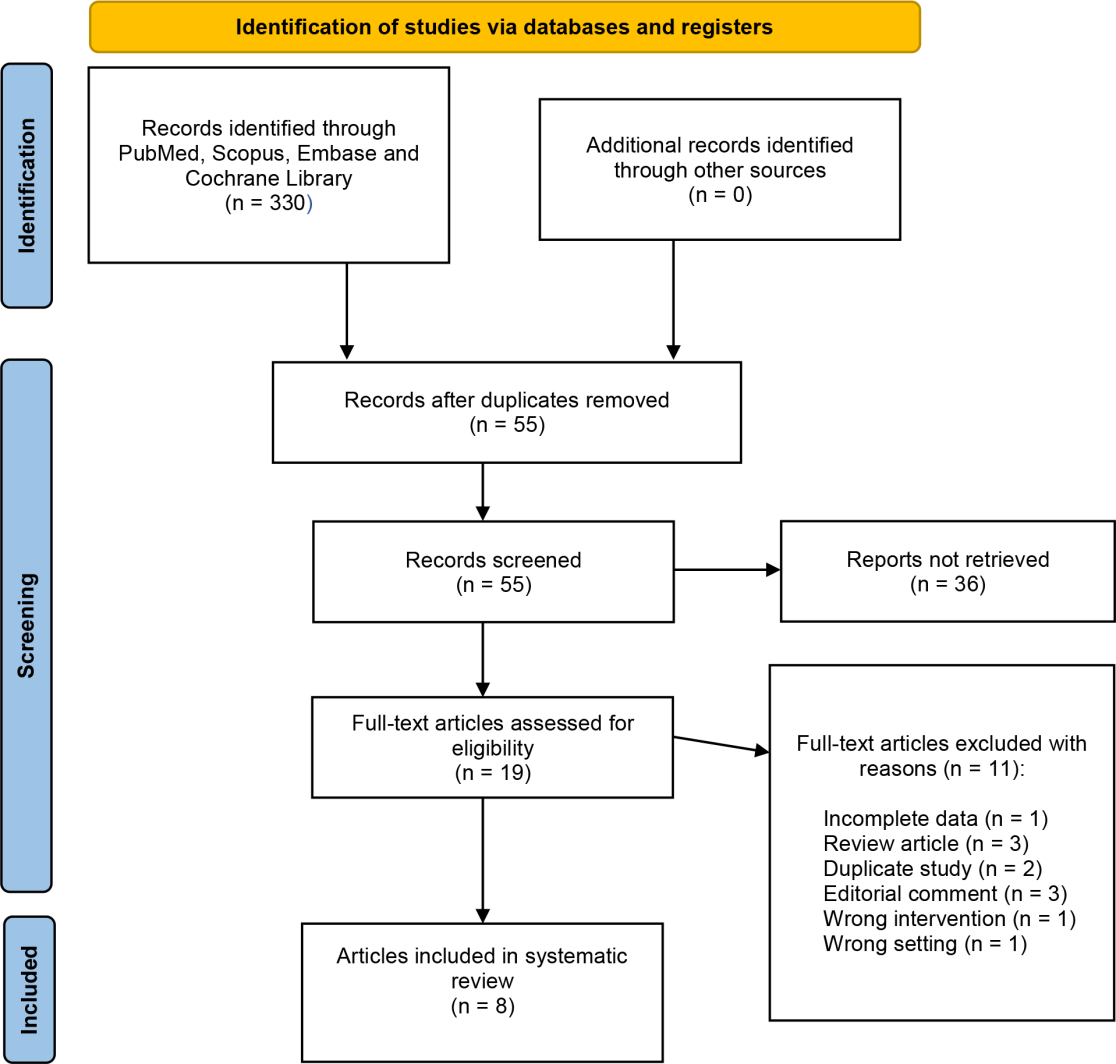
PRISMA flowchart.

The process of selecting studies is demonstrated by the PRISMA flowchart (**Figure 1**). After excluding duplicates and screening the abstracts and full texts, eight studies (5–7, 16–20) published between 2012 and 2022 were included for qualitative and quantitative analysis. The sample size comprised 1346 patients, of which 695 and 651 were treated with RPN and LPN, respectively. Three studies (7, 16, 20) were prospective nonrandomized studies, whereas the rest were observational retrospective case-control studies (5, 6, 17–19). Propensity matching analyzes were performed in four studies (6, 18–20). In addition, one study was a (7) multicenter study. **Table 1** provides an overview of the patient characteristics. There was no difference in terms of age (p = 0.62), male sex (p = 0.33), body mass index (BMI) (p = 0.21), tumor laterality (p = 0.69), CCI (p = 0.56), the American Society of Anesthesiologists (ASA) score (≥3) (p = 0.06), tumor size (p = 0.88), the RENAL score (p = 0.44), and preoperative estimated glomerular filtration rate (eGFR) (p = 0.56) (**Table 2**). Moreover, **Table S2** displays the tumor histological subtype, stage, and Furman grade.

**Table 1:** Overview of the included studies.

Study	Year	Country	Design	Technique	Patients	Number of surgeons	Score used	Surgical approach	Clamp method	Propensity scoring analysis	Median follow−up (months)	NOS	LE
Long	2012	USA	P, S	RPN	199	Multiple	RENAL ≥7	TP	NA	No	8.3	7	2b
				LPN	182								
Jang	2014	South Korea	R, S	RPN	89	Single	RENAL 7-10	NA	Selective clamp	No	NA	7	2b
				LPN	38								
Deng	2020	China	R, S	RPN	58	Single	RENAL ≥7	RP/TP	NA	Yes	39.5	7	2b
				LPN	58						31		
Wang	2016	China	R, S	RPN	81	Multiple	RENAL ≥7	RP/TP	NA	No	31.4	7	2b
				LPN	135						16.5		
Gu	2018	China	R, S	RPN	96	Multiple	RENAL ≥7	RP/TP	Total clamp	Yes	35	8	2b
				LPN	96						20.1		
Alimi	2018	France	P, M	RPN	50	NA	RENAL ≥7	TP	Unclamped mixed	No	19	7	2b
				LPN	50						14		
Zhang	2020	China	R, S	RPN	62	NA	RENAL ≥7	RP/TP	Unclamped mixed	Yes	25	8	2b
				LPN	62						25		
Garg	2022	India	P, S	RPN	60	Single	RENAL 10-12	TP	Total clamp	Yes	NA	8	2b
				LPN	30								

P, prospective; S, single center; R, retrospective; M, multiple center; RPN, robot-assisted partial nephrectomy; LPN, laparoscopic partial nephrectomy; RENAL, Radius, endophytic/exophytic, nearness, anterior/posterior location; TP, transperitoneal; RP, retroperitoneal; NA, not available; LE, level of evidence.

**Table 2:** Patients and tumor preoperative characteristics.

Variables	RPN vs LPN	I^2^(%)	*p*
Age WMD (95% CI)	-0.36 (-1.78 to 1.07)	17	0.62
Male OR (95% CI)	0.89 (0.71 to 1.12)	0	0.33
BMI WMD (95% CI)	0.54 (-0.31 to 1.39)	60	0.21
Right side OR (95% CI)	0.95 (0.74 to 1.22)	0	0.69
CCI WMD (95% CI)	-0.12 (-0.50 to 0.27)	76	0.56
ASA score (≥3) OR (95% CI)	3.07 (0.98 to 9.58)	75	0.06
Tumor size WMD (95% CI)	0.01 (-0.18 to 0.21)	68	0.88
RENAL score WMD (95% CI)	0.07 (-0.11 to 0.25)	76	0.44
Preoperative eGFR WMD (95% CI)	0.65 (-1.55 to 2.85)	55	0.56

LPN, laparoscopic partial nephrectomy; RPN, robotic partial nephrectomy; CCI, Charlson comorbidity index; ASA, American Society of Anesthesiologists; WMD, weighted mean difference; CI, confidence interval; OR, odds ratio; BMI, body mass index; eGFR, estimated glomerular filtration rate.

### 3.2 Surgical outcomes

**Figure 2:**
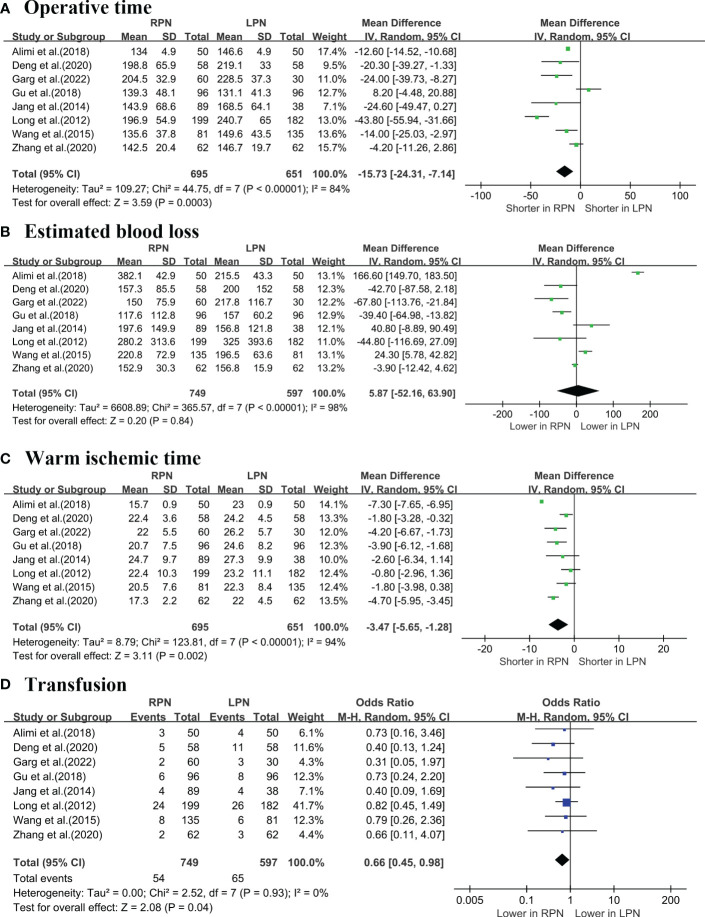
Forest plot comparison of RPN and LPN: **(A)** operative time; **(B)** estimated blood loss; **(C)** warm ischemic time; **(D)** transfusion.1 - PRISMA flowchart.

A cumulative of eight studies (5–7, 16–20) revealed that RPN exhibited a decreased operative time (OT) (WMD: -15.73 min; 95% CI: -24.31 to -7.14; p = 0.0003; **Figure 2A**), while the estimated blood loss (EBL) was similar for both techniques (WMD: 5.87 mL; 95% CI: -52.16 to 63.9; p = 0.84; **Figure 2B**). Furthermore, RPN had a shorter warm ischaemia time (WIT) (WMD: -3.47 min; 95% CI: -5.65 to -1.28; p = 0.002; **Figure 2C**) and lower transfusion rates compared with LPN (OR: 0.66; 95% CI: 0.45 to 0.98; p = 0.04; **Figure 2D**).

**Figure 3:**
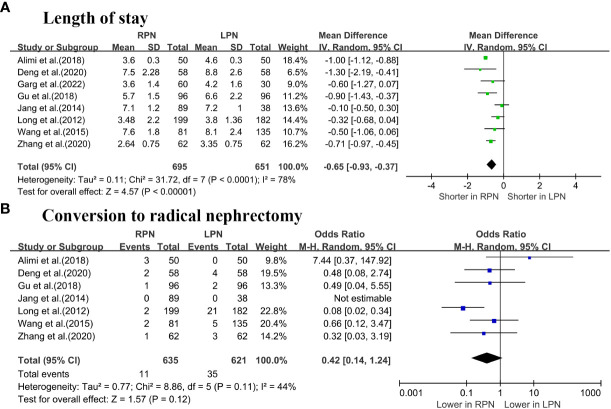
Forest plot comparison of RPN and LPN: **(A)** length of stay; **(B)** conversion to radical nephrectomy.

The length of stay (LOS) was significantly lower for RPN than LPN (WMD: -0.65 days; 95% CI: -0.93 to -0.37; p < 0.00001; **Figure 3A**) (5–7, 16–20). No differences were observed in terms of conversion to RN between the groups (OR: 0.42; 95% CI: 0.14 to 1.24; p = 0.12; **Figure 3B**) (5–7, 16–19).

### 3.3 Complications

**Figure 4:**
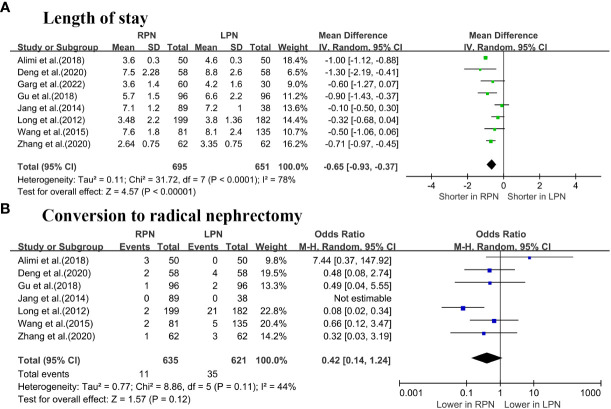
Forest plot comparison of RPN and LPN: **(A)** intraoperative complications; **(B)** postoperative complications; **(C)** major complications; **(D)** overall complications.

Intraoperative complication rates were 3.03% and 6.04% in the RPN and LPN groups, respectively (five studies; p = 0.04; **Figure 4A**) (5, 16–19). No significant differences were observed between the two groups regarding the postoperative complications (p = 0.11; **Figure 4B**) and the major (Clavien–Dindo ≥3) complication rate (p = 0.43; **Figure 4C**) (5–7, 16–20). The overall rates of complication in the RPN and LPN groups were 25.3% (149 out of 587 cases) and 28.9% (157 out of 543 cases), respectively (six studies; p = 0.15; **Figure 4D**) (5, 16–20).

### 3.4 Renal functional outcomes

**Figure 5:**
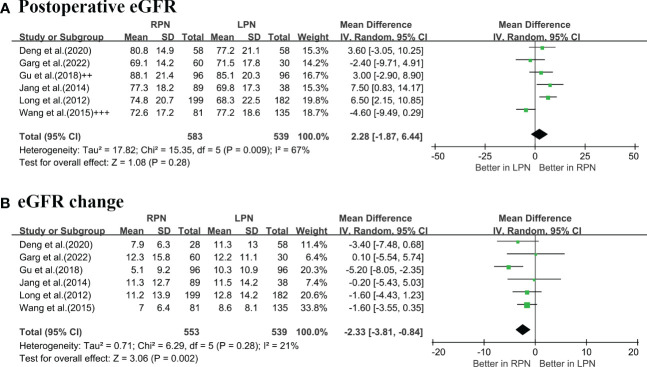
Forest plots of renal function: **(A)** postoperative eGFR; **(B)** eGFR change.

According to the pooled analysis of six studies (5, 6, 16–18, 20), the postoperative eGFR was similar between RPN and LPN (p = 0.28; **Figure 5A**); however, RPN was associated with a significantly lower degree of eGFR decline (OR: -2.33 mL/min/1.73 m^2^; 95% CI: -3.81 to -0.84; p = 0.002; **Figure 5B**).

### 3.5 Oncological outcomes

**Figure 6:**
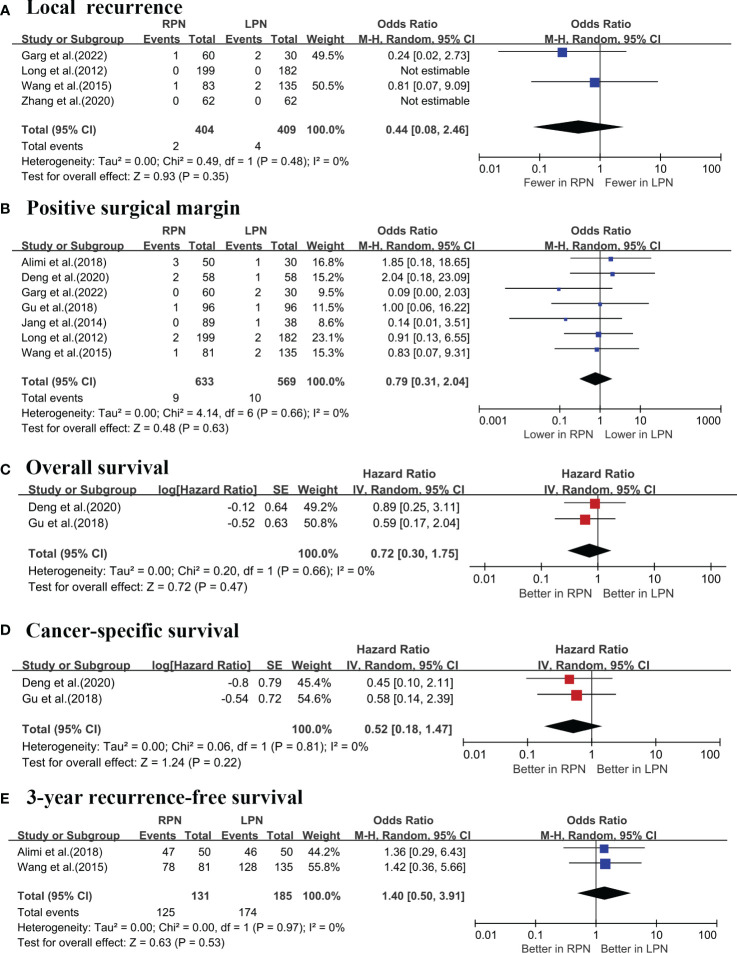
Forest plots of the oncological outcomes: **(A)** local recurrence; **(B)** positive surgical margin; **(C)** overall survival; **(D)** cancer-specific survival; **(E)** 3-year recurrence-free survival.

A cumulative analysis demonstrated that RPN and LPN produced similar results with respect to the local recurrence rates (four studies; p = 0.35; **Figure 6A**) (16, 17, 19, 20), Furthermore, there were no notable statistical differences in positive surgical margin (PSM) rates between the two procedures (seven studies; p = 0.63; **Figure 6B**) (5–7, 16–18, 20). Furthermore, no significant differences were observed in terms of the overall survival (OS) (two studies; HR = 0.72; p = 0.47; **Figure 6C**) (6, 18), cancer-specific survival (CSS) (two studies; HR = 0.52; p = 0.22; **Figure 6D**) (6, 18), and 3-year recurrence-free survival (RFS) (two studies; OR = 1.4; p = 0.53; **Figure 6E**) (7, 17).

### 3.6 Quality assessment

All included studies had a 2b level of evidence and moderate or high quality (**Table S3**). Additionally, the included studies demonstrated a moderate risk of bias (**Table S4**).

### 3.7 Heterogeneity

Our analysis revealed moderate to high heterogeneity levels (I^2^ > 60%) among studies in a few outcomes (OT, WIT, EBL and LOS and postoperative eGFR). However, attention should be paid to the significance bias introduced by small-sample studies on I^2^ statistic (21).

### 3.8 Sensitivity and subgroup analysis

A sensitivity analysis was used for the OT, WIT, EBL, LOS and postoperative eGFR to assess the reliability of our findings. Sensitivity analyses revealed that the results did not vary significantly after omitting one study by turn (**Figure S1**). The subgroup analysis revealed that the study design, country, and sample size contributed to varying degrees of heterogeneity between studies (**Table S5**). The country or region of the type of study was the primary source of heterogeneity in the operative time and the change in eGFR. Furthermore, differences in sample sizes contributed to heterogeneity in operative time, transfusion rate, RN conversion rate, and postoperative eGFR.

### 3.9 Publication bias

A comprehensive analysis was performed using a funnel plot to assess the likelihood of publication bias in OT, WIT, EBL and LOS. The funnel plot showed that the study distribution was relatively symmetrical; however, some indications of publication bias persisted.

## 4 Discussion

The ideal treatment of renal tumors should ensure satisfactory perioperative, functional, and oncological outcomes. Although robotic surgery offers better visual field and intraoperative stability, it is beneficial for renal anatomy and repair. The advantages of RPN compared to LPN in managing complex renal masses remain debatable (22).

Our pooled analysis revealed that RPN has a shorter OT, WIT compared with LPN. Although robotic surgery requires assistants to complete the docking and adjustment of the robotic arms, as well as re-docking and positioning when facing bilateral kidney tumors, technological advancements and the expertise of experienced operators in high-volume centers have resulted in significant improvements. In addition, the wide use of the early unclamping technique in the RPN group when faced with complex renal tumors results in higher intraoperative bleeding and a shorter WIT. While performing extracorporeal suturing within a limited WIT, the use of surgical robots provides significant advantages over laparoscopic forceps, including enhanced flexibility, superior visualization, and efficient fibrillation filtering (23). However, the subgroup analysis suggests that the study, country, and sample size might cause unavoidable heterogeneity.

Although the cumulative analysis revealed that RPN and LPN had comparable transfusion rates, the subgroup analysis revealed that RPN had an advantage over LPN in paired analyses (p = 0.03). RPN demonstrated a lower transfusion rate, albeit with consistent results limited to studies with smaller sample sizes (p = 0.04). The observed differences might not be solely attributed to the surgical approach, as technical variations and distinct clinical pathways between surgeons might also serve as contributing factors (7). It is noteworthy that the patients in the RPN group had a shorter hospital stay. However, potential influencing factors, such as hospital capacity levels, the primary surgeon’s preference for rapid recovery, and the national health insurance system, should be considered (5). Our cumulative analysis revealed that complex renal tumors were more likely to require conversion to RN during LPN compared to RPN, with rates of 5.6% and 1.7%, respectively. However, no statistical significance was observed.

Generally, complex tumors are often associated with higher complication rates. Tanagho et al. classified complication rates based on the RENAL score, with intraoperative complications occurring in 2.3%, 2.7%, and 8.2% of low, moderate, and high complexity renal masses (24), respectively. A multi-institutional study revealed an overall rate of complication of 15.8%, with 3.7% of patients experiencing major complications (25). Our cumulative results indicated lesser intraoperative complications occurred in RPNs compared with LPNs. However, Jang et al. (5) suggested that both approaches are more susceptible to intraoperative bleeding when dealing with complex renal tumors, particularly in the hilar region. Nonetheless, RPN three-dimensional visualization and operational stability provide some advantages. Interestingly, Loew’s meta-analysis of 4,919 patients revealed that RPN had lower postoperative complication rates compared with LPN (26). However, this advantage was not statistically significant when dealing with complex tumors, although RPN consistently exhibited a trend towards a reduced risk of complications.

Preservation of renal function is a primary objective of PN, and the quantity and quality of preserved nephrons are strongly associated with renal functional recovery after PN (27). Our findings indicate comparable postoperative eGFR levels between the RPN and LPN groups. However, subgroup analyses of small sample studies suggested higher postoperative eGFR levels for RPN. The potential confounding effects of a higher preoperative eGFR and a shorter median follow-up duration should be considered. Significantly smaller changes in eGFR in the RPN group were demonstrated by our pooled analysis. However, the multivariate linear regression model revealed that the surgical approach did not predict postoperative eGFR or the percentage change in eGFR, indicating similar functional outcomes for RPN and LPN (16). the timing of renal artery clamping might temporarily impact early eGFR (18, 28), while two randomized studies showed no significant difference in functional outcome in on-vs off-clamp RPN (29, 30). Furthermore, misinterpretation of renal functional outcomes is possible due to the compensatory function of the contralateral kidney (5).

The primary concern regarding the surgical approaches for renal tumors is the oncological outcomes. Our pooled analysis revealed the equivalence of oncological outcomes between RPN and LPN. PSM rates for both the RPN and LPN groups were 1.4 and 1.7%, respectively. Interim follow-up data showed no significant correlation between PSM patients and local recurrence after PN, and only active detection was required (31). Although the malignancy and complexity of the tumor could potentially influence PSM (32). No significant differences were observed in the OS, CSS and 3-yr RFS between RPN and LPN. Kizilay et al. reported that the surgical approach did not predict the 5-year CSS (33). Furthermore, different surgical approaches have similar oncologic outcomes (34).

Only one study directly compared the cost-effectiveness of RPN and LPN in the treatment of complex renal tumors, indicating that LPN is more advantageous in the control of hospitalization costs (17). Of course, the difference could be further reduced by controlling the length of hospital stay and operation time (35). in smaller centers, it is important to balance the costs and benefits of individualized surgical approaches and select the appropriate surgical plan for each patient. It is worth noting that three-dimensional (3D) virtual models have been shown to better perceive tumor depth and its relationship to intrarenal structures, thus more accurately assessing tumor complexity (36). For nephron sparing surgery for complex renal tumors, the aid of preoperative 3D model is conducive to the formulation of optimal surgical strategies. Campi et al. (37) achieved satisfactory results by using Hyperaccuracy 3D Virtual Models combined with RPN in the treatment of complex renal tumors with a horseshoe kidney. In the future, the use of 3D kidney models will further improve surgical procedures and outcomes.

## 5 Limitations

Firstly, the study included retrospective literature without any randomized controlled studies, resulting in low-quality evidence. Furthermore, no subgroup analysis was conducted to explore sources of heterogeneity based on surgical access (transperitoneal or retroperitoneal). Most procedures were performed in high-volume hospitals, and most patients who underwent RPN belonged to the latter half of the learning curve (the influence of experience). Finally, shorter follow-up durations and varying salvage and adjuvant treatments might impact the determination of prognostic tumor outcomes.

## 6 Conclusion

For individuals with complex renal tumors, RPN had similar functional and oncologic outcomes compared to LPN, with lower the OT, WIT, transfusion rate, LOS and intraoperative complications. Our findings need further validation in a large-sample prospective randomized study.

## Data availability statement

Articles/**Supplementary Material** provide all available data.

## Author contributions

Conceptualization and data curation: LW and CL. Methodology and software: LW and CL. Supervision: JD and LW; Writing draft and review: LW and P-YZ. All authors contributed to the article and approved the submitted version.

## Supplementary material

Supplementary Figure 1 Sensitivity analysis of perioperative outcomes.

Supplementary Figure 2 Publication bias testing.
