# Supplementary figures and images for: Perioperative, functional, and oncological outcomes of robotic vs. laparoscopic partial nephrectomy for complex renal tumors (RENAL score ≥7): an evidence-based analysis
Source: Front Oncol. 2023 Jun 2;13:1195910. doi: 10.3389/fonc.2023.1195910 (PMC10472455; doi:10.3389/fonc.2023.1195910)

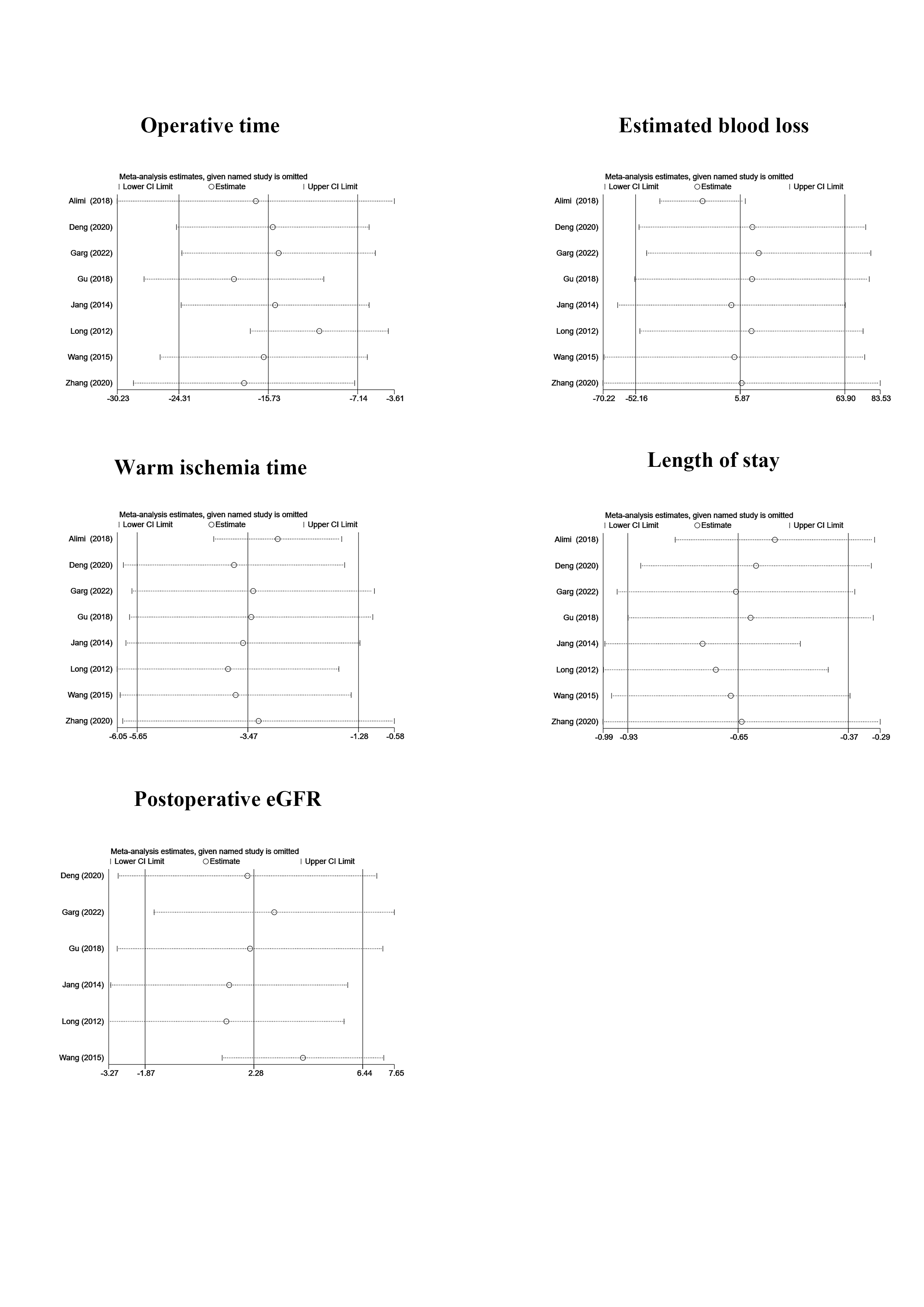

Supplement: Supplementary Figure 1 — Sensitivity analysis of perioperative outcomes. [file Image_1.tif]

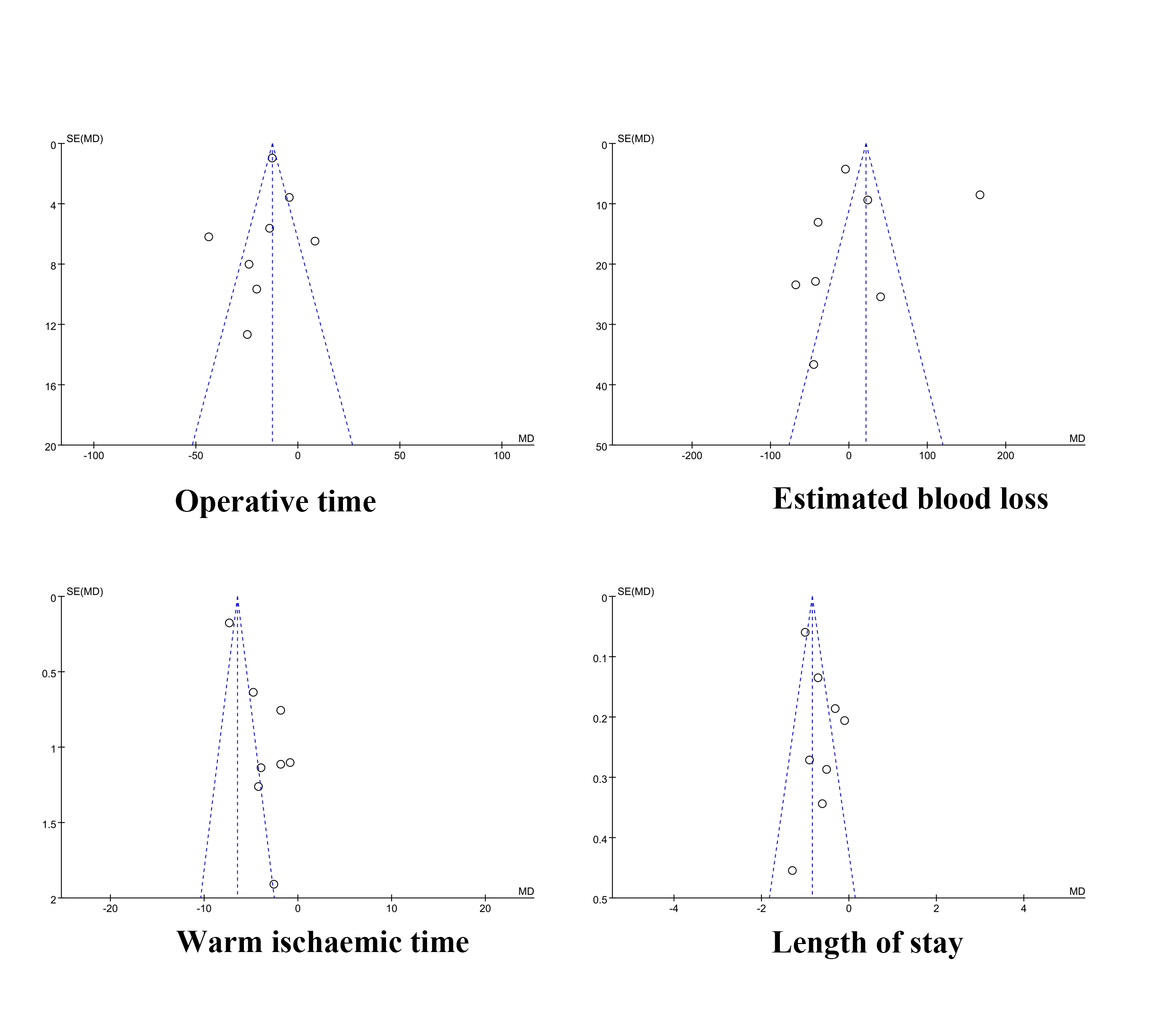

Supplement: Supplementary Figure 2 — Publication bias testing. [file Image_2.tif]
